# Preparation and Analysis of Sheath–Core Intelligent Thermo-Regulating Fiber

**DOI:** 10.3390/polym14091665

**Published:** 2022-04-20

**Authors:** Ronggen Zhang, Pei Feng, Chongchang Yang

**Affiliations:** 1 College of Mechanical Engineering, Donghua University, Shanghai 201620, China; rgzhang@mail.dhu.edu.cn (R.Z.); ycc@dhu.edu.cn (C.Y.); 2 Engineering Research Center of Advanced Textile Machinery, Donghua University, Shanghai 201620, China

**Keywords:** intelligent thermo-regulating fiber, phase-change material, sheath–core fiber, melt spinning

## Abstract

In this work, a skin–core composite intelligent temperature-adjusting fiber was prepared using the composite melt spinning method, with polypropylene as the skin layer and T28-type paraffin as the core layer, in order to obtain clothing fibers with a bidirectional temperature adjustment function. A differential scanning calorimeter was used to test the phase-change latent heat of fibers with different amounts of paraffin injections, and an infrared thermal imager was used to monitor the skin–core composite intelligent temperature-adjusting fiber bundles and ordinary polypropylene fiber bundles under the same heating and cooling conditions. The temperature of the fiber bundle was considered to be a function of time. The results showed that with the increase in the amount of the paraffin injections, the proportion of the paraffin component in the fiber and the latent heat of the fiber phase transition also increased. When the paraffin injection amount was 1.5 mL/min, the melting enthalpy and the crystallization enthalpy reached 65.93 J/g and 66.15 J/g, respectively. Under the same conditions, the heating speed of the intelligent temperature-adjusting fiber bundle was found to be slower than that of the ordinary polypropylene fibers, and the maximum temperature difference between the two reached 8.0 °C. Further, the cooling speed of the former was also observed to be slower than that of the latter, and the maximum temperature difference between the two reached 6.7 °C.

## 1. Introduction

With the improvements in people’s living standards and the upgrading of chemical fiber equipment, consumers have high requirements for fiber products. The requirements include comfortable, lightweight, and environmentally friendly fiber products. In recent years, relevant institutions in the textile industry have developed many fibers with special functions in order to meet people’s functional needs for chemical fiber products. These fibers are recognized by the market and appreciated by consumers for their excellent properties. Intelligent thermo-regulating fibers are one of the successful representatives [1].

Traditional wear textiles mainly rely on reducing or increasing the thickness of textiles in order to increase or decrease the heat exchange between the human body and the surrounding environment to maintain the normal temperature of the human body. When the ambient temperature is higher than the temperature of the human body, the heat dissipation of the human body is increased by reducing the thickness of the clothing, so that the human body can be kept cool. When the temperature of the external environment is lower than the temperature of the human body, the thickness of the clothes is increased to reduce the heat exchange between the human body and the environment, to achieve the purpose of keeping warm. However, maintaining a more comfortable temperature of the human body only by increasing or decreasing the thickness of the thermal insulation material is a passive temperature regulation method, which is not intelligent and cannot actively adapt to a cold or hot environment [2]. Therefore, fibers and textiles with intelligent thermo-regulating functions were introduced.

Intelligent thermo-regulating fibers are a high-tech product prepared by adding a phase-change material to a fiber using a special technology. It relies on the phase change of the phase-change material inside the fiber to absorb or release heat, realize the function of two-way temperature regulation, and form a relatively constant “microclimate environment” around the fiber [3]. Intelligent thermo-regulating textiles actively adjust the temperature of the human body according to the change in the external environment temperature and have certain intelligence [4].

At present, the preparation methods of the phase-change temperature-adjusting fibers mainly include the hollow fiber filling method and the spinning method. The hollow fiber filling method refers to filling the hollow part of the hollow fiber with PCMs in order to obtain a temperature-adjusting fiber. In the spinning method, the PCMs or phase-change microcapsules are added to the melt or spinning solution of the spinning polymer, and then the thermo-regulating fibers are processed by microcapsule spinning, composite spinning, or electrospinning [1,5].

Gao, T. et al. [6] demonstrate a personal thermal regulation textile based on thermally conductive and highly aligned BN/PVA fibers. The a-BN/PVA composite fibers are fabricated through a 3D printing technique followed by a hot-drawing treatment. As a result, the fabric based on the a-BN/PVA fibers has high thermal conductivity of 0.078 W/(m·K), which is 1.56 and 2.22 times higher than that of PVA fabrics and cotton fabrics, respectively. Zhao, L. et al. [7] fabricated ultrafine form-stable phase-change composite fibers consisting of n-octadecane and silk by the emulsion electrospinning method. Zhang, H. et al. [8] synthesized a series of microencapsulated PCMs (micro-PCMs) with good phase-change behavior through in-situ polymerization. The temperature of the board incorporated with 60 wt% micro-PCMs can be kept in the range of 22–27 °C for approximately 1735 s. Zhu, W. et al. [9] obtained a novel PEG/PVA composite PCF by electrospinning their aqueous solution instead of using an organic solvent, as in the traditional method. The CPCF-50 shows the optimal morphological structure and exhibits a relatively high latent heat of 72.3 J/g. The thermos-regulating times of heating and freezing processes of CPCF-50 are 59.0% and 89.5% longer than those of the control and CPCF-0, respectively. Abdalkarim, S.Y.H. et al. [10] prepared dipole responsive magnetic/solar-driven PCF composites reinforced with magnetic cellulose nanocrystal hybrids (MCNC). The obtained PCF/MCNC-5% showed excellent magnetic properties, with a saturation magnetization (MS) value of 1.3 emu/g, and effective latent heat phase-change enthalpies of 69.2 ± 3.5 J/g–83.1 ± 4.2 J/g. Tian, B. et al. [11] prepared thermally conductive form-stable phase-change materials (FSPCMs) by using ethylene-vinyl acetate (EVA), paraffin, expanded graphite (EG), and carbon fiber (CF) as row materials. FSPCMs displayed a melting temperature of 45.63 °C and latent heat of 167.4 J/g. The prepared material had a low leakage rate, which was less than 2%. Mengjin, J. et al. [12] prepared a new kind of thermo-regulating fiber based on polyvinyl alcohol (PVA) and paraffin by wet composite spinning. Results show that the thermo-regulating fiber has acceptable thermal stability when the content of paraffin in the fiber is less than 30%. The best fiber properties of tensile strength of 2.6 cN/dtex, breakage elongation of 19.9%, and latent heat of 24.45 J/g can be achieved by manipulating the preparation process, and the latent heat of the fiber still remains 24.32 J/g after washing 25 times. Pervez, M.N. et al. [13] used polyethylene glycol (PEG-1000) as PCM in an experiment and took a 2.5% concentration that was encapsulated by using an in-situ polymerization technique. The heat storage capacity of the 2.5% PEG-coated fabric was determined to be 2842.5120 J/g and for the binder-coated fabric 1557.8 J/g by DSC analysis, and FT-IR analysis of PEG microcapsules exhibited the highest peak at 3400–2400 cm^−1^. Shin, Y. et al. [14] prepared melamine–formaldehyde microcapsules containing eicosane by in-situ polymerization and developed thermo-regulating textile materials. The thermo-regulating fabrics had heat storage capacities of 0.91–4.44 J/g. Shim, H. et al. [15] quantified the effect of PCMS in clothing on heat flow from the body during temperature transients. The results indicate that the heating and cooling effects last approximately 15 min. Heat released by PCMS in a cold environment decreases body heat loss by an average of 6.5 W for a one-layer suit and 13.2 W for a two-layer suit compared with non-PCM counterparts. Lu, Y. et al. [16] achieved a material structure with high performance in overcoming leakage of PW by a coaxial electrospinning technique resulting from core–sheath structured smart textiles with PW as the core layer and polyacrylonitrile (PAN) as the sheath layer. It is noteworthy that the smart textile possesses high encapsulation efficiency of 54.3% (latent heat of 60.31 J/g) and shows good stability as there is almost no change in latent heat for the smart textiles after 500 heating–cooling cycles. Luo, D. et al. [17] fabricated a series of novel flexible phase-change smart lines by double encapsulating paraffin into polypropylene hollow fiber membranes (PPHFMs) and using expanded graphite (EG) to overcome liquid leakage during phase transition and enhance the thermal conductivity of paraffin. The results indicated that the thermal conductivity of PEP-CPCMs with 2.08 wt% EG was obviously improved by 75%, and the maximum paraffin encapsulation capacity was 85.31 wt%.

Presently, the production methods of intelligent temperature-adjusting fibers mainly include the filling method, fabric surface finishing method, spinning method, etc. The filling method is used to immerse the hollow fiber in the solution of the phase-change material, or use a vacuum pump to suck the solution of the phase-change material into the hollow cavity of the hollow fiber, and seal the end face of the hollow fiber after drying. The fabric finishing method is employed to coat or pad the phase-change material on the surface of the fabric. Using this method, the fabric obtains the function of temperature regulation. Further, when the intelligent temperature-adjusting fiber is prepared using the spinning method, the phase-change material is added to the solution or melt of the spinning polymer using a special method, and then spinning is performed in order to obtain the intelligent temperature-adjusting fiber containing the phase-change material. The commonly used composite spinning methods include the solution spinning and the melt spinning methods. The preparation of intelligent temperature-regulating fibers using melt spinning has many advantages, such as the simple process, low pollution, and low consumption cost.

Chen, C. et al. [18] fabricated ultrafine phase-change fibers (PCFs) with a core–sheath structure based on polyethylene glycol/cellulose acetate (PEG/CA) blends successfully via coaxial electrospinning for thermal energy storage. The results from DSC demonstrated that the composite fibers imparted balanced and reversible phase-change behaviors. Lu, Y. et al. [19] obtained green phase-change nanofiber mats with a core–sheath structure based on paraffin wax emulsion (PWE) and polyvinyl alcohol (PVA) for thermal energy storage (TES) by the coaxial electrospinning approach. The core–sheath structured PWE@PVA nanofibers mats have high ΔHm of 48.25 J/g and freezing enthalpy (ΔHc) of −47.75 J/g. Furthermore, only a slightly decreasing tendency in the enthalpy can be observed after 500 heating–cooling cycles, demonstrating good TES stability and the practicability of the as-prepared PWE@PVA nanofiber mats. Wen, G.-Q. et al. [20] developed a facile and controllable microfluidic strategy to fabricate core–shell phase-change microfibers with high paraffin Rubitherm^®^27 (RT27) content. The maximum melting enthalpy and crystallization enthalpy of the microfibers are approximately 128.2 J g^−1^ and 124.0 J g^−1^, respectively, and the corresponding encapsulation ratio is as high as 70%. Yi, L. et al. [21] fabricated core–sheath structured nanofibers with polyvinyl butyral (PVB) as the sheath and octadecane as the core by melt coaxial electrospinning. Pure octadecane without any solvents was used as the core solution; thus, the optimal sample possessed very high latent heat up to 118 J g^−1^. Lu, Y. et al. [22] explored a facile approach to obtain PW-loaded core–sheath structured flexible nanofiber films via the coaxial electrospinning technique. The PW as the core layer was successfully encapsulated by the sheath-layer poly(methyl methacrylate).The core–sheath nanofiber films, moreover, possessed the highest latent heat of 58.25 J/g and solidifying enthalpy of –56.49 J/g. Wang, S. et al. [23] used coaxial electrospinning to prepare core–sheath nanofibers loaded with phase-change materials (PCMs). The latent heat of nanofibers prepared using this sheath solution could be as high as 137.05 J/g. In the meantime, the properties of nanofibers in terms of thermal decomposition, mechanical strength, and elongation rate were also improved. Iqbal, K. et al. [24] reported a type of smart monofilament fiber development incorporated with microencapsulated phase-change material through a melt spinning process. Up to 12% microcapsules are successfully incorporated into the polypropylene monofilament, showing 9.2 J/g of latent heat. Van Do, C. et al. [25] fabricated polyethylene glycol (PEG)/polyvinylidene fluoride (PVDF) core–shell nanofibers by using coaxial electrospinning. In the core–shell composite nanofibers, melted PEG and PVDF solutions were coaxially electrospun (e-spun) through a double spinneret as a core layer and as a shell layer, respectively. In terms of energy storage capacity, core–shell nanofibers, fabricated at the core feed rate of 0.210 mL/h, had the largest content of PEG in the core of up to 42.5 wt%, with a latent heat of 68 J/g and a melting temperature of 62.8 °C. Naeimirad, M. et al. [26] summarized the current progress in the preparation of core–sheath fibers by melt spinning methods, and summarized the factors affecting the formation of core–sheath fibers. It was considered that, for polymers with a low melting point, it is suitable to prepare core–sheath fibers using the melt spinning method. In this work, paraffin was used as the phase-change material, which has a low melting point. Therefore, the method of direct composite spinning of phase-change materials and spinning polymers was used to prepare intelligent temperature-regulating fibers. By improving the traditional melt spinning equipment, the low-temperature phase-change material was directly delivered to the spinning pack with nitrogen as the pressure source. The phase-change material need not pass through the high-temperature and high-pressure areas such as the screw and metering pump. Therefore, it is not affected by the shearing action of the screw and the high-pressure conditions. Further, this shortens the residence time of the phase-change material in the high-temperature area and reduces the relative phase change of the high-temperature and high-pressure conditions in the melt spinning process. The adverse effects of the variable materials were found to be beneficial for the preparation of intelligent thermo-regulating fibers with better thermo-regulating properties.

## 2. Numerical Simulation

**Figure 1:**
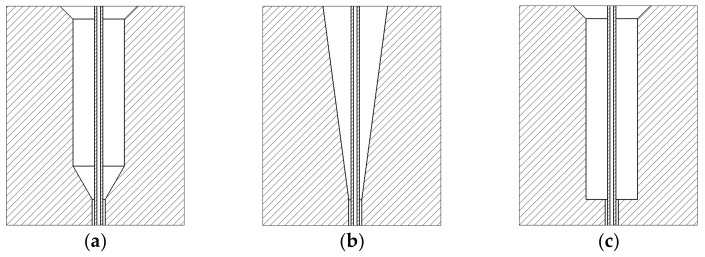
Three types of guide holes (**a**) Cylindrical guide hole. (**b**) Conical guide hole. (**c**) Flat-bottomed guide hole.

The role of the guide hole is to continuously and smoothly guide the melt into the micro-hole. Usually, the guide hole structures of the spinneret holes are cylindrical, conical, and flat-bottomed. In this work, for the sheath–core intelligent thermo-regulating fiber spun, the liquid paraffin and polypropylene melt were combined in the spinneret orifice, but only met at the exit of the spinneret’s micro-holes. Therefore, a conduit concentric of the guide hole has to be installed in the spinneret hole. Further, a catheter was used to introduce the paraffin into the core layer of the fiber. Three different guide hole configurations with the catheter are shown in Figure 1.

### 2.1. Modeling

**Figure 2:**
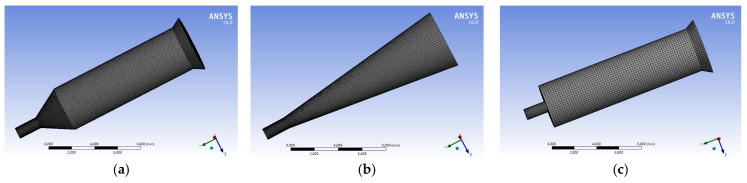
Three models of spinneret guide holes. (**a**) Cylindrical guide hole. (**b**) Conical guide hole. (**c**) Flat-bottomed guide hole.

In order to study the influence of the guide hole structure on the melt fluidity, the flow process of the melt in the spinneret hole was numerically simulated using the POLYFLOW software. The velocity distribution, pressure distribution, and the shear rate distribution of the melt in the spinneret channel with three different guide hole structures, namely the cylindrical guide hole, conical guide hole, and the flat-bottom guide hole, were obtained. The three-dimensional models of the spinneret orifice with three different guide hole structures were established, imported into the POLYFLOW software, and then meshed for the spinneret orifice model, as shown in Figure 2.

### 2.2. Material Parameters

The polypropylene melt characteristic parameters used in the simulation are shown in Table 1.

### 2.3. Boundary Conditions

The inlet boundary conditions are that the single-hole flow q is 2.8 × 10^−8^ m^3^/s at the inlet of the spinneret, and the temperature is 210 °C.

The wall boundary conditions are as follows: set the melt and the inner wall of the component without slippage, i.e., the normal velocity Vn = 0, the tangential velocity Vs = 0, and the wall temperature and box temperature are both 210 °C.

The outlet boundary condition is that the outlet is set to outflow.

The iterative methods used were Picard iteration for the melt viscosity, linear iteration for the pressure, and mini-element iteration for the velocity.

## 3. Experimental Methods

The experimental methods for the preparation of sheath–core intelligent thermo-regulating fibers, and the performance characterization tests, are discussed below.

### 3.1. Preparation of Sheath–Core Intelligent Thermo-Regulating Fiber

During the preparation of sheath–core intelligent thermo-regulating fibers, paraffin was fed into the spin pack by hot nitrogen gas. Then, polypropylene chips were passed through the single screw extruder (Zhejiang Jinhu Group, Zhoushan City, Zhejiang Province, China. D = 30 mm, L/D = 25), melted under the set spinning temperature, and transported to the metering pump, and then flowed into the spinning assembly after being accurately metered using a metering pump. Thereafter, the two melts were extruded through the spinning assembly and then compounded to form a skin–core compound intelligent temperature-regulating fiber. The flow of paraffin was 0.9 mL/min, 1.2 mL/min, and 1.5 mL/min, respectively. The mass percentage of paraffin in the fiber was approximately 34.9%, 41.7%, and 47.2%, respectively. The diameter of the prepared fibers in the experiments was 82 μm, 97 μm and 108 μm, respectively. The spinning process parameters that were set up in the experiment are shown in Table 2.

### 3.2. Performance Characterization Test

The performance characterization test included the thermal performance test and the thermo-regulating test. They are briefly discussed below.

#### 3.2.1. Thermal Performance Test

A differential scanning calorimeter (TA Instruments, USA; model: Q20) was used to test the phase transition latent heat of skin–core composite intelligent temperature-regulating fibers injected with different amounts of paraffin and ordinary polypropylene fibers without paraffin. The test was performed under a N2 environment with a gas flow of 50 mL/min. During the test, the temperature was increased from 0 °C to 55 °C at a heating rate of 10 °C/min, and then cooled from 55 °C to 0 °C at a cooling rate of 10 °C/min.

#### 3.2.2. Thermo-Regulating Performance Test

In order to conduct the thermo-regulating performance test, 35 intelligent temperature-adjusting fibers and ordinary polypropylene fibers were cut with a length of 250 mm. Then, the 35 fibers were wound together and both ends were fixed with fixators in order to produce intelligent temperature-adjusting fiber bundles and polypropylene fiber bundles. The heating device was placed vertically and the temperature was kept constant after the heating device was heated to 42.0 °C. The two fiber bundles were cooled to 22.0 °C and placed close to the front of the CNC heating block. The FLIR A615 infrared thermal imager was used to monitor the temperature of the two fiber bundles in real time.

During the exothermic test, the two fiber bundle samples were heated to 35 °C, and then the two fiber bundles were placed in an ambient condition with an average temperature of 15.8 °C, allowing them to cool down freely, and a thermal imager was used to monitor the temperature changes of the fiber bundles in real time.

Due to the uneven temperature distribution of each area on the heating block, a marking line was made on the heating block before the experiment. The line was placed as the base. The temperature of each area on the fiber bundle was also different during the heating process, so the average temperature along the centerline of the fiber bundle was used to characterize the temperature of the fiber bundle.

## 4. Results and Discussion

The results of the numerical simulations, thermal performance, and thermo-regulating performance are discussed below.

### 4.1. Numerical Simulation Results

#### 4.1.1. Distribution of Velocity

**Figure 3:**
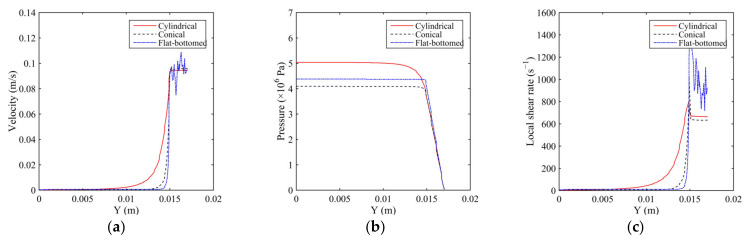
The velocity, pressure, and shear rate distribution of the melt along the axis. (**a**) Velocity distribution. (**b**) Pressure distribution. (**c**) Shear rate distribution.

Figure 3 shows a graph showing the velocity change of the melt in the spinneret hole with three different guide hole structures along the axis of the spinneret hole. From the figure, it is observed that the change in speed is the smoothest, and the melt flow is more uniform. Further, it is observed that the melt flows from the cylindrical guide hole spinneret with a conical transition angle to the conical transition zone of the channel, its speed begins to increase, and the flow reaches the micropore. Moreover, the velocity of the melt in the flat-bottomed guide hole spinneret is found to have little change in the guide hole, and the melt velocity changes abruptly when it flows to the micro-hole. Furthermore, the velocity change gradient is the largest compared with the other two guide hole structures. It is also found that the melt velocity in the micropore fluctuates significantly.

**Figure 4:**
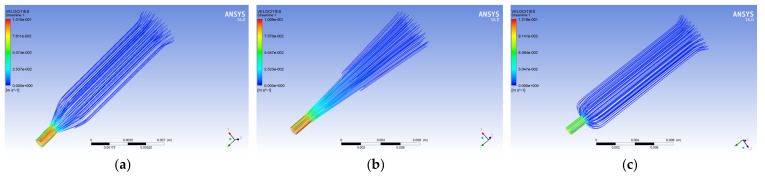
Streamline diagram of melt velocity in different guide holes. (**a**) Cylindrical guide hole. (**b**) Conical guide hole. (**c**) Flat-bottomed guide hole.

Figure 4 shows the flow diagram of the melt velocity in the spinneret hole of the three different guide holes. From the figure, it is observed that the polypropylene melt flows slowly at the guide hole with a larger diameter. The velocity of the melt increases gradually, and the velocity reaches the maximum when it flows into the pores. Further, by observing the melt velocity cloud diagram on the outlet surface of the micropore, it is found that the melt flow near the wall of the micropore and the outer wall of the conduit is slower, while the melt velocity at the center of the wall of the micropore and the outer wall of the conduit is the highest.

#### 4.1.2. Distribution of Pressure

**Figure 5:**
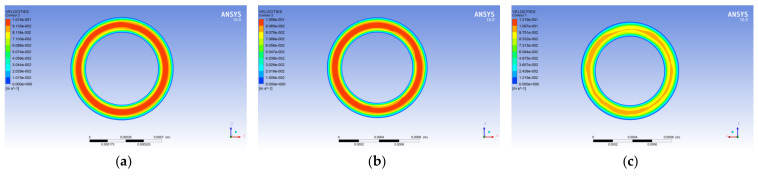
Velocity nephogram at the exit surface of the micropore with different guide holes. (**a**) Cylindrical guide hole. (**b**) Conical guide hole. (**c**) Flat-bottomed guide hole.

Figure 5 shows a graph showing the pressure change of the melt in the spinneret hole along the axis of the spinneret holes for the three different guide hole structures. From Figure 5, it is observed that the pressure change of the melt in the spinneret with the tapered guide hole along the axis direction is the highest. Further, it is gentle, and the pressure gradient of the melt along the axis direction in the flat-bottomed guide hole spinneret channel is the largest.

**Figure 6:**
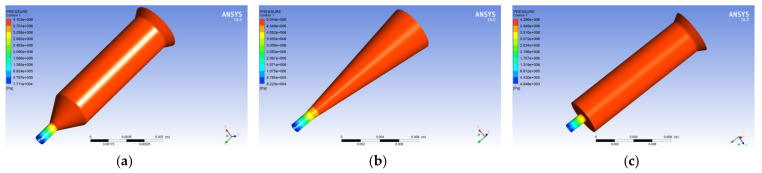
Cloud map of melt pressure distribution in different pilot holes. (**a**) Cylindrical guide hole. (**b**) Conical guide hole. (**c**) Flat-bottomed guide hole.

Figure 6 shows the pressure distribution cloud map of the melt in the spinneret with the three different guide hole structures. From the pressure cloud map, it is found that the pressure of the melt flowing to the micropore changes significantly, and the spinneret hole with the three different guide hole structures is the largest. The pressure drops are 4.103 MPa, 5.044 MPa, and 4.386 MPa, respectively, which shows that when the same flow of polypropylene melt flows through the spinneret orifice, the extruding force required by the spinneret hole of the tapered guide hole is the largest, while spinneret holes with cylindrical pilot holes require the lowest amount of squeezing force.

#### 4.1.3. Distribution of Shear Rate

**Figure 7:**
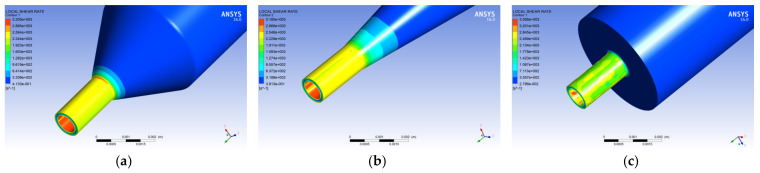
Cloud map of melt shear rate distribution in different pilot holes. (**a**) Cylindrical guide hole. (**b**) Conical guide hole. (**c**) Flat-bottomed guide hole.

Figure 7 illustrates the graph showing the shear rate variation curve of the melt in the spinneret hole along the axis of the spinneret hole with the three different guide hole structures. It is observed that the change value in the spinneret hole of the tapered guide hole is the smallest, and the change value in the spinneret hole of the flat-bottomed guide hole is the largest. Further, the shear rate of the melt in the orifice of the orifice spinneret along the axis of the spinneret is the gentlest, while the change in the shear rate of the melt in the channel of the flat-bottomed spinneret is the largest, and the melt shears in the micro-hole. The rate also fluctuates significantly.

**Figure 8:**
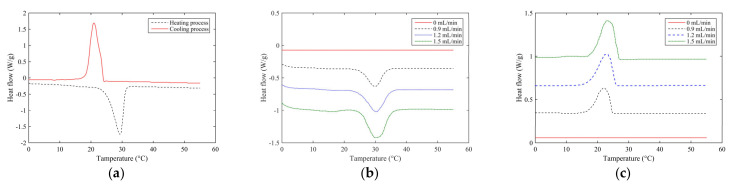
DSC curves of 100% paraffin and thermo-regulating fibers with different paraffin flow rates in heating and cooling process. (**a**) Heating and cooling process of paraffin. (**b**) Heating process of fiber. (**c**) Cooling process of fiber.

Figure 8 shows the distribution cloud diagram of the shear rate of polypropylene melt in the spinneret with different hole structures. Observing the melt shear rate distribution in the figure, it is found that the three different hole structures are cylindrical, conical, and flat-bottomed. Further, the maximum shear rates of the melt in the spinneret holes are 3205 s^−1^, 3185 s^−1^, and 3556 s^−1^, respectively, and the maximum shear rate of the melt flow occurs at the wall of the conduit near the microporous area. The shear rate distribution of the melt at the micro-holes of the tapered and cylindrical orifice spinnerets is found to be more uniform than that at the micro-holes of the flat-bottomed orifice spinneret.

From the results, it is found that when compared to the cylindrical guide hole and the flat-bottomed guide hole, the flow velocity, pressure, and shear rate of the melt in the conical guide hole along the axis direction change more smoothly. Moreover, in the spinneret orifice, the flow velocity and the shear rate of the melt are found to fluctuate minimally, which is more favorable for the fiber extrusion. Therefore, the conical guide hole was chosen to produce the fibers.

### 4.2. Thermal Performance

Figure 8 shows the DSC curves of 100% paraffin and thermo-regulating fibers with different paraffin flow rates in the heating and cooling process. It can be seen from the curve that the paraffin is in a melting and endothermic process in the temperature range of 26–33 °C. With the increase in temperature, paraffin gradually changes from solid to liquid, accompanied by energy absorption and storage. However, in the temperature range of 24.5 °C to 16 °C, the crystallization process is exothermic. With the decrease in temperature, the paraffin gradually changes from liquid to solid, and the stored energy is released. It is found that when the temperature range is between 25.79 °C and 36.38 °C, the DSC curve of the polypropylene fibers without paraffin injection does not change; the DSC curve of the paraffin-infused fibers showed a melting peak in this temperature range, indicating that when the temperature reaches this range, the paraffin wax inside the fiber undergoes a solid–liquid phase transition and absorbs heat, thereby keeping the external ambient temperature unchanged or slowing down the temperature. Further, it is observed that when the temperature range was between 25.85 °C and 14.78 °C, the DSC curve of the polypropylene fiber without paraffin did not change significantly, while the DSC curve of the paraffin-infused fiber showed a crystallization peak in this temperature range. This shows that in this temperature range, as the external temperature decreases, the paraffin inside the fiber undergoes a liquid–solid phase transition and releases heat, thereby keeping the external ambient temperature unchanged or slowing down the decrease in temperature.

Comparing the heating and cooling process of 100% paraffin and thermo-regulating fibers with different paraffin flow rates, it can be seen that the melting peak and crystallization peak of 100% paraffin are the highest, indicating that the melting enthalpy and crystallization enthalpy of the fiber are mainly due to the role of paraffin; in other words, the thermal regulation is mainly contributed by paraffin. Furthermore, comparing the heating and cooling DSC curves of the fibers injected with different amounts of paraffin, it is found that the shapes of the melting peaks and crystallization peaks of the three fibers are similar, but with the increase in the amount of paraffin injection, the areas contained in the melting peaks and crystallization peaks of the fibers increase. The results show that the melting enthalpy and crystallization enthalpy of the fibers have increased. The heating and cooling DSC curves of the fibers were analyzed, and the melting and crystallization performance parameters of the fibers were obtained, as shown in Table 3.

### 4.3. Thermo-Regulating Performance

**Figure 9:**
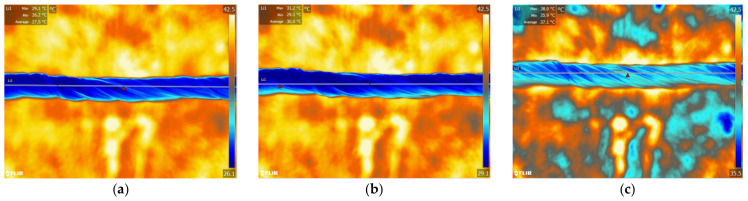
Infrared thermal imaging images of intelligent temperature-regulating fiber bundles at different heating times. (**a**) t = 30 s. (**b**) t = 80 s. (**c**) t = 130 s.

**Figure 10:**
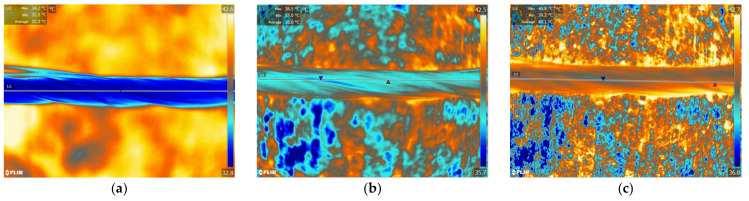
Infrared thermal images of common polypropylene fiber bundles at different heating times. (**a**) t = 30 s. (**b**) t = 80 s. (**c**) t = 130 s.

**Figure 11:**
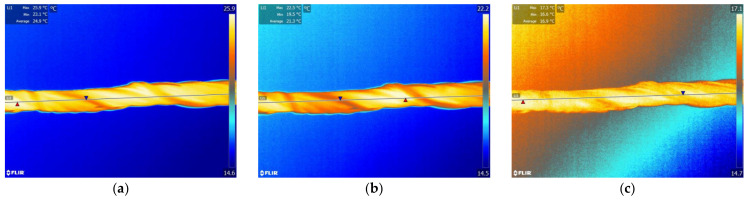
Infrared thermal images of intelligent temperature-regulating fiber bundles at different cooling times. (**a**) t = 40 s. (**b**) t = 155 s. (**c**) t = 220 s.

**Figure 12:**
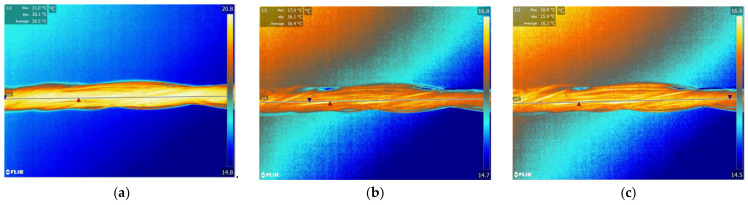
Infrared thermal images of ordinary polypropylene fiber bundles in different cooling time. (**a**) t = 40 s. (**b**) t = 155 s. (**c**) t = 220 s.

**Figure 13:**
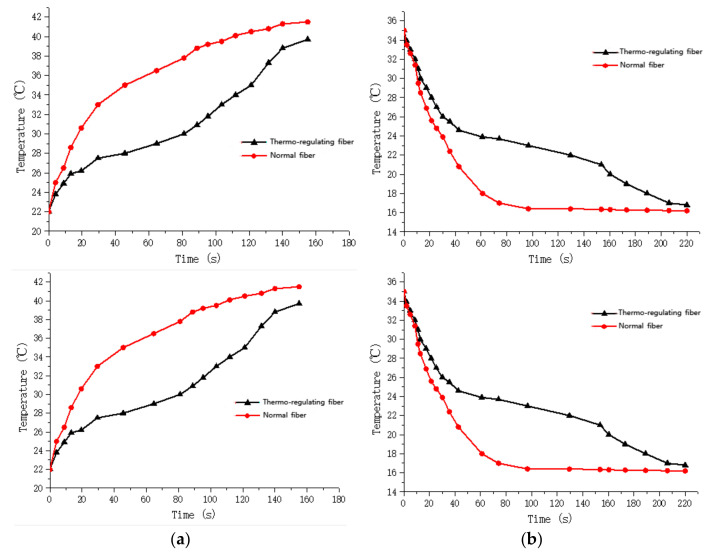
Temperature changes with time for fiber bundles in endothermic and exothermic tests. (**a**) Endothermic test. (**b**) Exothermic test.

Figure 9 and Figure 10 show the thermographic images of the intelligent temperature-adjusting fiber bundles and the ordinary polypropylene fiber bundles at different heating and cooling times, respectively. Figure 11 and Figure 12 show the thermographic images of the intelligent temperature-adjusting fiber bundles and the ordinary polypropylene fiber bundles at different cooling times, respectively. Figure 13 shows the variation in the average temperature along the centerline of the two fiber bundles with time during the heating and cooling processes. From Figure 13a, it is found that the heating rate of the ordinary polypropylene fiber bundles shows a trend of fast first and then slow, while the heating rate of intelligent temperature-adjusting fiber bundles shows a trend of first fast, then slow, and then fast. Further, the temperature of the bundle is found to rise rapidly in the time interval of t = 15–120 s; the phase-change material in the fiber undergoes a solid–liquid phase transition and is accompanied by heat absorption and storage, thereby delaying the heating rate of the fiber. From 120 s to 155 s, the phase-change material in the fiber was liquefied, and the heating rate of the fiber was found to gradually increase. Under the same heating conditions and the same heating time, the temperature of the intelligent temperature-adjusting fiber bundle is lower than that of the polypropylene fiber bundle, and the maximum temperature difference between the two fiber bundles reaches 8.0 °C.

The temperature change trend of the two fiber bundles during the cooling process is found to be similar to the temperature change trend during the heating process. The cooling rate of the ordinary polypropylene fibers is first fast and then slow, while the temperature of the intelligent temperature-adjusting fiber bundle decreases rapidly in the first 40 s, and during the phase transition of the fiber bundle within the time interval of t = 40–160 s, the material undergoes a liquid–solid phase change and is accompanied by the release of heat, thereby delaying the cooling rate of the fiber bundle. During the time interval of t = 160 s to 220 s, the phase-change material in the fiber had solidified, and the cooling rate of the fiber bundle is found to be increased. Further, under the same cooling conditions and the same cooling time, the average temperature along the centerline of the intelligent temperature-adjusting fiber bundle is higher than that of the polypropylene fiber bundle, and the maximum temperature difference between the two fiber bundles reaches 6.7 °C.

## 5. Conclusions

When the temperature range was between 25.79 °C and 36.38 °C, the DSC curve of the polypropylene fiber without paraffin injection did not change, while the DSC curve of the fiber injected with paraffin wax showed a melting peak in this temperature range, indicating that when the temperature at this range is reached, the paraffin wax inside the fiber would undergo a solid–liquid phase transition to absorb heat, thereby keeping the external ambient temperature unchanged or slowing down the temperature rise.

When the temperature range was between 14.78 °C and 25.85 °C, the DSC curve of the polypropylene fiber without paraffin injection did not change, while the DSC curve of the fiber injected with paraffin wax shows a crystallization peak in this temperature range, indicating that in this temperature range, as the external temperature decreases, the paraffin inside the fiber undergoes liquid–solid phase conversion and releases heat, thereby keeping the external ambient temperature unchanged or slowing down the temperature decrease.

The heating rate of the ordinary polypropylene fiber bundles showed a trend of first fast and then slow, while the heating rate of intelligent temperature-adjusting fiber bundles showed a trend of first fast, then slow, and then faster. The temperature of the fiber bundles increased within the first 13 s of heating. Further, during the time interval of 15 s to 120 s, the phase-change material in the fiber underwent solid–liquid phase transition and was accompanied by heat absorption and storage, thereby delaying the heating rate of the fiber. During the time interval of 120 s to 155 s, the phase-change material in the fiber had liquefied, and the heating rate of the fiber had increased. Under the same heating conditions and the same heating time, the temperature of the intelligent temperature-adjusting fiber bundle was lower than that of the polypropylene fiber bundle, and the maximum temperature difference between the two fiber bundles reached 8.0 °C.

The cooling rate of the ordinary polypropylene fibers was first fast and then slow, while the temperature of the intelligent temperature-adjusting fiber bundle decreased rapidly in the first 40 s, and the phase-change material inside the fiber bundle occurs within the time interval of 40 s to 160 s. The liquid–solid phase transition was accompanied by the release of heat, thereby delaying the cooling rate of the fiber bundle. During the time interval of 160 s to 220 s, the phase-change material in the fiber had solidified, and the cooling rate of the fiber bundle had increased. Under the same cooling conditions and the same cooling time, the average temperature along the centerline of the intelligent temperature-adjusting fiber bundle was higher than that of the polypropylene fiber bundle, and the maximum temperature difference between the two fiber bundles reached 6.7 °C.

## Figures and Tables

**Table 1:** Characteristic parameters of polypropylene melt.

Material	Polypropylene Melt
Non-Newtonian exponents (n)	0.46
Relaxation time (s)	0.003
Zero shear viscosity (Pa·s)	115
Density (kg/m^3^)	900
Thermal conductivity (w/m/k)	0.21
Heat capacity (J · kg/k)	2550
Spinning temperature (°C)	210

**Table 2:** Spinning process parameters of skin–core composite intelligent temperature-regulating fibers.

Items		Process Parameters
Screw extruder	Zone 1 (°C)	190
	Zone 2 (°C)	195
	Zone 3 (°C)	200
	Zone 4 (°C)	205
Melt temperature before the pump (°C)		208
Melt temperature after pump (°C)		210
Spin pack temperature (°C)		210
Paraffin temperature (°C)		50
Flow rate of polypropylene melt (m^3^/s)		2.8 × 10^−8^
Paraffin delivery pressure (MPa)		0.08
Cooling air temperature (°C)		15

**Table 3:** Parameters of melting and crystallization properties of fibers prepared with different paraffin injection amounts.

Paraffin Flow (mL/min)	Melting Process				Crystallization Process			
	Tp (°C)	ΔHm (J/g)	Onset (°C)	Endset (°C)	Tp (°C)	ΔHc (J/g)	Onset (°C)	Endset (°C)
0.9	29.89	43.37	25.79	34.85	22.12	44.86	24.75	14.78
1.2	30.26	54.13	26.22	35.76	22.52	55.59	25.21	15.55
1.5	31.02	65.93	26.53	36.38	23.16	66.15	25.85	16.28

## Data Availability

The data presented in this study are available on request from the corresponding author.
